# Association of the time course of Chinese visceral adiposity index accumulation with cardiovascular events in patients with hypertension

**DOI:** 10.1186/s12944-023-01852-w

**Published:** 2023-07-01

**Authors:** Yuntao Wu, Wenqi Xu, Lu Guo, Wenjuan Li, Lisha Zhang, Lishu Gao, Chenrui Zhu, Shuohua Chen, Liming Lin, Shouling Wu

**Affiliations:** 1 Department of Cardiology, Kailuan General Hospital, Tangshan, 063000 China; 2 Graduate School, North China University of Science and Technology, Tangshan, China; 3 Department of Endocrinology, Tangshan People’s Hospital, Tangshan, 063000 Hebei China

**Keywords:** Chinese visceral adiposity index, Cumulative measurement, Time course, Adverse cardiovascular event, Visceral fat

## Abstract

**Background:**

The Chinese visceral adiposity index (CVAI), a simple surrogate measure of visceral fat, is significantly associated with cardiovascular disease (CVD) risk in the general population. This study aimed to evaluate the association of cumulative CVAI (cumCVAI) exposure and its accumulation time course with CVD risk among patients with hypertension.

**Methods:**

This prospective study involved 15,350 patients with hypertension from the Kailuan Study who were evaluated at least three times in the observation period of 2006 to 2014 (2006–2007, 2010–2011, and 2014–2015) and who were free of myocardial infarction and stroke before 2014. The cumCVAI was calculated as the weighted sum of the mean CVAI for each time interval (value × time). The time course of CVAI accumulation was categorized by splitting the overall accumulation into early (cumCVAI_06 − 10_) and late (cumCVAI_10 − 14_) accumulation, or the slope of CVAI versus time from 2006 to 2014 into positive and negative.

**Results:**

During the 6.59-year follow-up period, 1,184 new-onset CVD events were recorded. After adjusting for confounding variables, the hazard ratios (HRs) and 95% confidence intervals (CIs) for CVD were 1.35 (1.13–1.61) in the highest quartile of cumCVAI, 1.35 (1.14–1.61) in the highest quartile of the time-weighted average CVAI, 1.26 (1.12–1.43) in those with a cumulative burden > 0, and 1.43 (1.14–1.78) for the group with a 10-year exposure duration. When considering the time course of CVAI accumulation, the HR (95% CI) for CVD was 1.33 (1.11–1.59) for early cumCVAI. When considering the combined effect of cumCVAI accumulation and its time course, the HR (95% CI) for CVD was 1.22 (1.03–1.46) for cumCVAI ≥ median with a positive slope.

**Conclusions:**

In this study, incident CVD risk depended on both long-term high cumCVAI exposure and the duration of high CVAI exposure among patients with hypertension. Early CVAI accumulation resulted in a greater risk increase than later CVAI accumulation, emphasizing the importance of optimal CVAI control in early life.

**Supplementary Information:**

The online version contains supplementary material available at 10.1186/s12944-023-01852-w.

## Background

Cardiovascular disease (CVD) remains the most prominent cause of mortality and permanent disability worldwide [1, 2]. Stroke affected more than 523 million people in 2019 and accounts for 18.6 million CVD-related deaths annually [1]. Existing evidence suggests that overweight and hypertension are independent risk factors for CVD [3]. An umbrella review of 12 systematic reviews and 53 meta-analyses [4] suggested that being overweight (high body mass index [BMI]) contributed to 4 million deaths in 2015, over two thirds of which were attributable to CVD. Additionally, with every 5 kg/m^2^ increase in BMI, the risk of CVD increases by 10%. Fuchs et al. [5] found that a blood pressure of > 115/75 mmHg (1 mmHg = 0.133 kPa) combined with each incremental increase in blood pressure of 20/10 mmHg is associated with an approximately two-fold increase in CVD risk. A previous study showed that in contrast to a single risk factor, being overweight combined with the presence of hypertension puts individuals at an increased risk of CVD development [6]. Considering that overweight and hypertension tend to co-exist, early detection and prevention, as well as control measures, for both risk factors could confer cardiovascular benefit.

Both basic experiments and interventional trials have demonstrated that visceral obesity is a determinant risk factor for adverse CVD-related events in individuals who are classed as overweight [7]. However, although there are uniform standards for blood pressure measurement, the gold-standard techniques for assessing visceral obesity, namely magnetic resonance imaging (MRI), bioelectrical impedance analysis, and computed tomography (CT), are limited in clinical practice and in epidemiological promotion due to their technical complexity, high cost, and potential risk of radiation exposure [8, 9]. Moreover, simple indices, such as the waist-to-hip ratio and waist circumference (WC), cannot be used to accurately assess the amount of visceral fat [10].

The Chinese visceral adiposity index (CVAI), which combines traditional indicators with lipid parameters, is a novel surrogate index that shows high concordance with the validation results of CT [11–13]. Wan et al. [14] showed that measuring the CVAI could improve CVD risk prediction compared with traditional indices. However, previous studies have been confined to a single time point measurement of CVAI, which is insufficient to accurately reflect the longitudinal effect of cumulative CVAI on CVD risk over time.

In this prospective cohort study of the Chinese population, this research is the first to explore the association between the time course of CVAI accumulation and future CVD among patients with hypertension.

## Methods

### Study design and population

The city of Tangshan had approximately 7.72 million inhabitants in 2022, and its demographic composition, urban and rural distribution, industrial layout, and per capita gross domestic product are similar to those at the national level. The city is situated approximately 150 km from southeast Beijing. Therefore, socioeconomically, the population of this study can be regarded as being representative, at least to some extent.

The detailed methodology and study design have been described previously [14, 15]. At its simplest, 101,510 employees (81,110 men; age range 18–98 years) from the Kailuan Corporation underwent baseline examination, with follow-up every 2 years thereafter. At follow-up, questionnaires were completed to collect participant information on lifestyle, demographics, disease history, and drug history, as well as anthropometric measurements and biochemical indicators. The annual occurrence of CVD was verified by reviewing the diagnostic records and medical insurance system of the Kailuan Group.

**Fig. 1:**
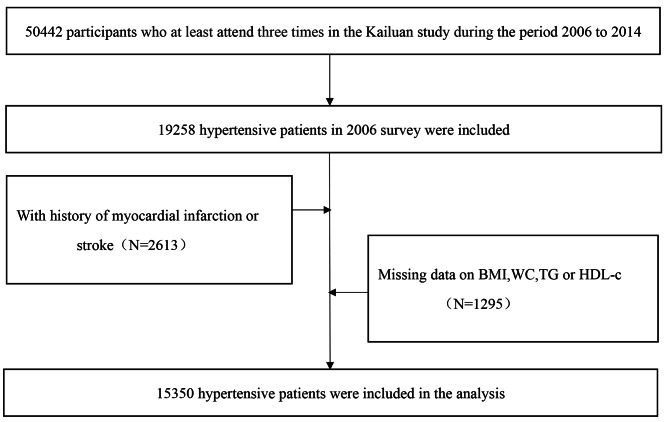
Flow chart

The long-term cumulative CVAI was determined from the data of 19,258 patients with hypertension who were evaluated at least three times during the period between 2006 and 2014 (2006–2007, 2010–2011, and 2014–2015). Among these patients, 2,613 patients with a history of myocardial infarction (MI) and stroke before 2014 were excluded, and 1,295 patients with missing data for triglyceride (TG), high-density lipoprotein cholesterol (HDL-c), WC, or BMI before 2014 were excluded. Therefore, 15,350 patients with hypertension were included in the analysis (Fig. 1). All of the participants provided written informed consent, and the study protocol was approved by the ethics committee of Kailuan General Hospital.

### Calculation of cumulative CVAI (cumCVAI), time-weighted average (TWA)-cumCVAI, cumulative burden, duration of high CVAI, and early versus late cumCVAI

**1. CVAI was calculated as follows** [14]:

CVAI (Female) = 39.76 × Lg [fasting TG (mmol/L)] + 1.71 × age + 4.32 × BMI + 1.12 × WC (cm) − 11.66 × fasting HDL-c (mmol/L) − 187.32.

CVAI (Male) = 22.00 × Lg [fasting TG (mmol/L)] + 0.68 × age + 0.03 × BMI + 4.00 × WC. (cm) − 16.32 × fasting HDL-c (mmol/L) − 267.93.

**2. CumCVAI was calculated as follows** [16]:

CumCVAI = [(CVAI_06_ + CVAI_08_)  /2 × (time_1–2_)] + [(CVAI_08_ + CVAI_10_) / 2 × (time_2–3_)] + … + [(CVAI_12_ + CVAI_14_)  /2 × (time_4–5_).

where CVAI_06_, CVAI_08_, CVAI_10_, CVAI_12_, and CVAI_14_ represent the CVAI at the first, second, third, fourth, and fifth examinations, and time_1–2_, time_2–3_, time_3–4_, and time_4–5_ indicate the time intervals between consecutive visits in years. The means of time_1–2_, time_2–3_, time_3–4_, and time_4–5_ were 2.06, 1.95, 2.26, and 2.11 years, respectively.

**3. TWA-cumCVAI was calculated as follows** [17]:

TWA-cumCVAI = cumCVAI/(time_1–5_), where time_1–5_ indicates the total measurement period.

**4. The cumulative burden was calculated as follows** [18]:

Cumulative burden = [(CVAI_06_ + CVAI_08_) / 2 − cutoff value] × (time_1–2_) + [(CVAI_08_ + CVAI_10_)/ 2 − cutoff value] × (time_2–3_) + … + [(CVAI_12_ + CVAI_14_) /2 − cutoff value × (time_4–5_)].

Currently, there is no recognized cutoff value for CVAI. This research determined the cutoff value for the receiver operating characteristic curve (110.6) using R software (www.r-project.org). The value was considered 0 when the values between two consecutive examinations were < 0, as described previously [19].

**5. Duration of high CVAI was calculated as follows:**

High CVAI was defined as CVAI values of ≥ 110.6. The duration of high CVAI was determined as the number of visits with high CVAI values across the five checks, classified as 0 years (a high CVAI value never occurred), 2 years (a high value occurred once), 4 years (a high value occurred twice), 6 years (a high value occurred three times), 8 years (a high value occurred four times), and 10 years (a high value occurred at all five examinations) [20].

**6. The time course of CVAI accumulation was calculated as follows** [21]:

The time course of CVAI accumulation was split into two, as follows: the cumCVAI between 2006 and 2010 (cumCVAI_06–10_) and the cumCVAI between 2010 and 2014 (cumCVAI_10–14_), indicating early and late CVAI exposure, respectively. Additionally, the slope of CVAI versus time from 2006 to 2014 was calculated using the least-squares principle and linear regression, where a negative or positive slope indicated a decrease or increase in CVAI over time.

### Data collection and definitions

Information on demographics (sex, age, and educational attainment), lifestyle variables (physical activity, alcohol consumption, and smoking), and clinical characteristics (medication history and disease history) was collected via questionnaires. Physical examinations included measurements of WC, weight, and height. Weight was accurate to 0.1 kg, and height was accurate to 1 mm. BMI was calculated as body mass (kg)/ height (m)^2^ [22, 23]. Physical exercise was classified as “never or sometimes” or “often.” Smoking was classified as “never or previous smoker” or “current smoker.” Drinking was classified as “never or previous drinker” or “current drinker.” Hypertension was defined on the basis of either a history of hypertension (self-reported), treatment with anti-hypertensive medications, or a blood pressure of ≥ 140/90 mmHg [24]. Diabetes mellitus was defined as a history of diabetes mellitus diagnosed by a doctor (self-reported), the use of hypoglycemic drugs, or a fasting blood glucose (FBG) of ≥ 7.0 mmol/L [25].

For the laboratory examinations, venous blood was collected in the morning after the participant had fasted for at least 8 h. The sera from all participants were analyzed by centralized testing at Kailuan General Hospital using a Hitachi automated biochemical analyzer (Hitachi 747; Hitachi, Tokyo, Japan). Triacylglycerol (TG) was determined using the glycerol-phosphate oxidase-peroxidase coupling method. Low-density lipoprotein cholesterol (LDL-c) and HDL-c were determined using the direct method, and total cholesterol (TC) was determined using the TC oxidase method.

### Outcome assessment

The primary endpoint was major adverse cardiovascular events during follow-up, namely MI and stroke. The start of follow-up was the date of the 2014 annual health examination, and individuals were followed up until 31 December 2021. The diagnoses of CVDs were based on the codes from the 10th Revision of the International Classification of Diseases (hemorrhagic stroke: I60–I61; ischemic stroke: I63; MI: I21) [26, 27]. A database of CVD diagnoses, which was updated annually throughout the follow-up period, was created using data from the Municipal Social Insurance Institution. Endpoint event adjudication was performed by trained medical personnel using abstracted information from inpatient medical records and medical insurance data.

### Statistical analysis

SAS 9.4 software (SAS Institute, Cary, NC, US) was used for the statistical analyses. Categorical variables were compared using the chi-square test, while continuous variables were compared using the Kruskal–Wallis test or analysis of variance on the basis of whether the data were normally distributed. A *P* value of < 0.05 was considered statistically significant.

The incidence density (cases per 1,000 person-years) and accumulated incidence were measured. Kaplan–Meier analyses were used to calculate the cumulative incidences of CVDs of different categories of CVAI indicators, and the log-rank test was used for statistical comparisons. Hazard ratios (HRs) and 95% confidence intervals (CIs) were evaluated by multivariate Cox proportional hazards regression models between CVDs and cumCVAI indicators in the following categories: (i) cumCVAI (quartile 1: <267,892.09 as the reference group); (ii) TWA-cumCVAI (quartile 1: <88.03 as the reference group); (iii) cumulative burden (cumulative burden = 0 as the reference group); and (iv) when the combined effect of CVAI accumulation and its time course was considered, the subjects were split into four groups on the basis of the direction of the slope (negative or positive) and the median cumCVAI (332,983.38). Adjustments were made for sex and age in Model (1) Adjustments were made for current smoker, current alcohol drinker, physical activity, education level, sex, and age in Model (2) Further adjustments were made for FBG; estimated glomerular filtration rate (eGFR); LDL-c; high-sensitivity C-reactive protein (hs-CRP); systolic blood pressure (SBP); and use of lipid-lowering drugs, hypoglycemic drugs, and anti-hypertensive drugs in Model (3) This research tested the proportional hazards assumption using Schoenfeld residuals.

To evaluate the impact of cumCVAI-associated indicators by category on CVD risk, we performed hierarchical analyses by sex (male vs. female), normal blood pressure attainment status (yes vs. no), and age (< 60 vs. ≥ 60 years) as exploratory analyses. Furthermore, to verify the robustness of the results, we performed several validation analyses. Specifically, Fine–Gray competing risk models were used, considering non-CVD death as a competing event. Moreover, to avoid reverse causality, we excluded subjects who developed CVDs with a follow-up duration of less than the first year. This research also excluded participants who did not undergo five consecutive examinations from 2006 to 2014, participants with a history of hepatitis, and participants with data in repeat analyses. We also used the net reclassification index (NRI), C-index, and integrated discrimination improvement (IDI) to evaluate the improvement in the prediction performance of the CVAI and cumCVAI for CVD beyond traditional risk factors.

## Results

### Baseline characteristics

Of the 15,350 eligible subjects included in the analysis, the mean age was 58.92 ± 10.65 years, and 82.42% of the participants were male. The baseline characteristics of the participants according to the joint effect of cumCVAI and its time course are shown in Table 1. Compared with the group with cumCVAI < median and slope < 0, subjects in the other groups were more likely to have higher LDL-c, TG, TC, SBP, BMI, hs-CRP, diastolic blood pressure (DBP), and FBG values, as well as a higher prevalence of lipid-lowering drug, anti-diabetic drug, and anti-hypertensive drug use, and diabetes mellitus.

**Table 1:** Baseline characteristics of the participants stratified by cumCVAI and CVAI slope

Characteristics	Overall	CumCVAI < median, Slope < 0	CumCVAI < median, Slope ≥ 0	CumCVAI ≥ median, Slope < 0	CumCVAI ≥ median, Slope ≥ 0	*P*
Participants	15,350	2758	4917	2567	5108	
Age, years	58.92 ± 10.65	57.93 ± 10.04	55.51 ± 9.89	63.19 ± 10.51	60.59 ± 10.64	< 0.01
Male, N (%)	12,561(82.42)	2463(89.30)	3959(80.52)	2191(85.35)	4038(79.05)	< 0.01
SBP, mmHg	147.66 ± 19.38	146.82 ± 19.95	144.71 ± 18.59	150.43 ± 19.59	149.55 ± 19.29	< 0.01
DBP, mmHg	89.38 ± 10.51	88.49 ± 10.61	88.22 ± 10.27	90.51 ± 10.72	90.42 ± 10.41	< 0.01
BMI, kg/m²	26.03 ± 7.60	24.22 ± 6.89	24.74 ± 8.14	27.01 ± 5.16	27.75 ± 8.01	< 0.01
FBG, mmol/L	5.77 ± 1.53	5.74 ± 1.59	5.51 ± 1.23	6.13 ± 1.78	5.85 ± 1.57	< 0.01
TC, mmol/L	5.35 ± 1.04	5.29 ± 1.00	5.34 ± 1.02	5.29 ± 1.02	5.42 ± 1.09	< 0.01
LDL-C, mmol/L	2.97 ± 0.83	2.97 ± 0.83	2.98 ± 0.82	2.96 ± 0.81	2.97 ± 0.84	0.74
HDL-C, mmol/L	1.33 ± 0.34	1.46 ± 0.37	1.39 ± 0.34	1.26 ± 0.30	1.23 ± 0.29	< 0.01
TG, mmol/L	1.41(0.98–2.11)	1.15(0.81–1.66)	1.29(0.91–1.91)	1.44(1.02–2.17)	1.69(1.19–2.47)	< 0.01
hs-CRP, mg/L	1.04(0.51–2.30)	0.93(0.50-2.00)	0.92(0.50-2.00)	1.20(0.60–2.70)	1.20(0.51–2.70)	< 0.01
eGFR, [mL/(min·1.73²)]	95.68 (83.76–105.10)	98.41 (88.37-107.05)	99.40 (88.96-107.99)	90.40 (77.71–99.89)	92.94 (80.19-102.53)	< 0.01
Current smoking, N (%)	5994(39.05)	1235(44.78)	2077(42.24)	903(35.18)	1779(34.83)	< 0.01
Current drinking, N (%)	4697(30.60)	850(30.82)	1551(31.54)	770(30.00)	1526(29.87)	0.28
Physical exercisers, N (%)	12,256(79.84)	2254(81.73)	3969(80.72)	1957(76.24)	4076(79.80)	< 0.01
High school or above	2670(17.39)	427(15.48)	904(18.39)	440(17.14)	899(17.60)	< 0.01
	**Overall**	**CumCVAI<median, Slope<0**	**CumCVAI<median, Slope ≥ 0**	**CumCVAI ≥ median, Slope<0**	**CumCVAI ≥ median, Slope ≥ 0**	***P***
Salt level, > 10 g/d	13,520(88.08)	2434(88.25)	4271(86.86)	2282(88.90)	4533(88.74)	< 0.01
Diabetes, N (%)	2792(18.19)	407(14.76)	584(11.88)	652(25.40)	1149(22.49)	< 0.01
Antidiabetic agents, N (%)	1251(8.15)	174(6.31)	201(4.09)	367(14.30)	509(9.96)	< 0.01
Antihypertensive agents, N (%)	6252(40.73)	942(34.16)	1684(34.25)	1268(49.40)	2358(46.16)	< 0.01
Lipid-lowering agents, N (%)	563(3.67)	67(2.43)	106(2.16)	125(4.87)	265(5.19)	< 0.01

P, comparison of baseline characteristics between different groups

SBP systolic blood pressure, DBP diastolic blood pressure, BMI body mass index, FBG fasting blood glucose, TC total cholesterol, LDL-C low-density lipoprotein cholesterol, HDL-C high-density lipoprotein cholesterol, hs-CRP high-sensitivity C reactive protein, TG triglyceride, eGFR estimated glomerular filtration rate. CVAI, Chinese visceral adiposity index;

### Association of cumCVAI, TWA-cumCVAI, cumulative burden, and duration of high CVAI with CVD risk

During the follow-up period of up to 6.54 years (2015–2021), 1,184 new-onset CVD events were documented (355 cases of MI, 800 cases of stroke, and 29 cases of combined MI and stroke). Additionally, the incidence density and cumulative incidence rate for CVD were 12.46 per 1,000 person-years and 7.71%, respectively. Table 2 shows the incidence density of CVD and the multivariate HRs for the cumCVAI parameters. The incidence density of CVD per 1,000 person-years increased gradually according to the quartiles of cumCVAI, ranging from 8.23 (95% CI 7.17–9.44) in quartile 1 to 16.03 (95% CI 14.46–17.76) in quartile 4. Additionally, the fully adjusted HR (95% CI) was significantly higher in quartile 2 [1.21 (1.01–1.45)], quartile 3 [1.29 (1.07–1.54)], and quartile 4 [1.35 (1.13–1.61)] than in quartile 1. To examine whether the association was affected by the single measurement of CVAI, we additionally adjusted for CVAI in 2006 and 2014 on the basis of the above models, respectively. The trend in the analysis of the effect of TWA-cumCVAI on CVD risk was consistent with that of cumCVAI (Supplementary Table S1).

**Table 2:** Hazard ratios and 95% confidence intervals for CVD risk stratified by cumulative CVAI indicators

Index	Case, n (%)	Incidence rate^a^	Model 1	Model 2	Model 3
CumCVAI					
**Quartile 1**	204(5.32)	8.23(7.17–9.44)	Reference	Reference	Reference
**Quartile 2**	278(7.24)	11.55(10.27–12.99)	1.29(1.08–1.55)	1.30(1.09–1.56)	1.21(1.01–1.45)
**Quartile 3**	337(8.78)	14.40(12.94–16.03)	1.49(1.25–1.78)	1.51(1.27–1.80)	1.29(1.07–1.54)
**Quartile 4**	365(9.51)	16.03(14.46–17.76)	1.52(1.28–1.82)	1.54(1.29–1.84)	1.35(1.13–1.61)
***P*** **for trend**			< 0.01	< 0.01	< 0.01
**TWA-CVAI**					
**Quartile 1**	213(5.55)	8.73(7.63–9.98)	Reference	Reference	Reference
**Quartile 2**	259(6.75)	10.82(9.58–12.22)	1.17(0.97–1.40)	1.17(0.98–1.41)	1.09(0.91–1.31)
**Quartile 3**	346(9.02)	14.73(13.25–16.36)	1.50(1.26–1.78)	1.52(1.28–1.80)	1.28(1.07–1.52)
**Quartile 4**	366(9.54)	15.79(14.25–17.49)	1.51(1.27–1.79)	1.52(1.28–1.81)	1.35(1.14–1.61)
***P*** **for trend**			< 0.01	< 0.01	< 0.01
**Cumulative burden**					
**= 0**	491(6.21)	9.86(9.02–10.77)	Reference	Reference	Reference
**> 0**	693(9.32)	15.32(14.22–16.51)	1.40(1.24–1.57)	1.41(1.25–1.58)	1.26(1.12–1.43)
**Time exposure duration**					
**0 year**	166(5.07)	7.95(6.83–9.25)	Reference	Reference	Reference
**2 years**	204(7.00)	11.23(9.79–12.88)	1.26(1.03–1.55)	1.27(1.03–1.56)	1.21(0.99–1.49)
**4 years**	189(7.41)	11.99(10.40-13.82)	1.28(1.04–1.58)	1.29(1.05–1.59)	1.18(0.95–1.45)
**6 years**	230(8.93)	14.63(12.86–16.65)	1.54(1.26–1.88)	1.55(1.27–1.90)	1.39(1.13–1.70)
**Index**	**Case, n (%)**	**Incidence rate** ^**a**^	**Model 1**	**Model 2**	**Model 3**
**8 years**	228(9.71)	16.08(14.12–18.31)	1.66(1.35–2.03)	1.67(1.36–2.05)	1.42(1.15–1.74)
**10 years**	167(9.92)	16.20(13.92–18.85)	1.71(1.37–2.12)	1.73(1.39–2.15)	1.43(1.14–1.78)
***P*** **for trend**			< 0.01	< 0.01	< 0.01
**Combination of CumCVAI and CVAI slope**					
**CumCVAI<median, Slope<0**	186(6.74)	10.75(9.31–12.41)	Reference	Reference	Reference
**CumCVAI<median, Slope ≥ 0**	296(6.02)	9.38(8.37–10.51)	0.98(0.81–1.18)	0.98(0.82–1.18)	1.01(0.84–1.21)
**CumCVAI ≥ median, Slope<0**	240(9.35)	14.96(13.65–16.38)	1.24(1.03–1.51)	1.25(1.03–1.52)	1.12(0.92–1.36)
**CumCVAI ≥ median, Slope ≥ 0**	462(9.04)	15.70(13.83–17.82)	1.32(1.11–1.56)	1.33(1.12–1.58)	1.22(1.03–1.46)
***P*** **for trend**			< 0.01	< 0.01	< 0.01

Model 1: adjusted for age and sex;

Model 2: adjusted for age, sex, smoking status, drinking status, education, and physical activity;

Model 3: further adjusted for SBP, eGFR, hs-CRP, FBG, LDL-c, Antidiabetic agents, Antihypertensive agents, Lipid-lowering agents;

Incidence rate^a^: per 1000 person-years

Cut off value of CVAI = 110.6; Median of cumulative CVAI was 332983.38

Similar trends were seen for cumCVAI and high CVAI duration. For those with a cumulative burden > 0, the CVD risk increased by 126% (HR 1.26, 95% CI 1.12–1.43) compared with a cumulative burden ≤ 0. Compared with participants in the group with no high CVAI values (0 years), the HRs (95% CIs) for CVD risk were 1.21 (0.99–1.49) in the 2-year group, 1.18 (0.95–1.45) in the 4-year group, 1.39 (1.13–1.70) in the 6-year group, 1.42 (1.15–1.74) in the 8-year group, and 1.43 (1.14–1.78) in the 10-year group.

### Early vs. late cumCVAI measurement and CVD risk

The periods of cumCVAI measurements were evaluated by dividing the overall observation period (2006–2014) into early (cumSUA_06 − 08_) and late (cumSUA_08–10_) periods (Table 3**).** Compared with the lowest group (quartile 1), the HRs (95% CIs) for the highest group (quartile 4) in the early and late periods were 1.33 (1.11–1.59) and 1.24 (1.04–1.49), respectively.

**Table 3:** Association of the time course of CVAI accumulation with CVD risk

	Quartile 1	Quartile 2	Quartile 3	Quartile 4	*P* for trend
CumCVAI_06 − 10_					
**Model. 1**	Reference	1.29(1.07–1.55)	1.49(1.25–1.79)	1.53(1.28–1.83)	0.01
**Model. 2**	Reference	1.30(1.08–1.56)	1.51(1.26–1.80)	1.55(1.30–1.86)	0.01
**Model. 3**	Reference	1.20(1.00-1.45)	1.26(1.04–1.51)	1.33(1.11–1.59)	0.01
**CumCVAI**_**10 − 14**_					
**Model. 1**	Reference	1.25(1.05–1.49)	1.42(1.19–1.68)	1.44(1.21–1.71)	0.01
**Model. 2**	Reference	1.26(1.06–1.50)	1.43(1.21–1.70)	1.45(1.22–1.72)	0.01
**Model. 3** ^**a**^	Reference	1.16(0.97–1.39)	1.18(0.96–1.44)	1.24(1.04–1.49)	0.01

Model 1: adjusted for age and sex;

Model 2: adjusted for age, sex, smoking status, drinking status, education, and physical activity;

Model 3: further adjusted for SBP, eGFR, hs-CRP, FBG, LDL-c, Antidiabetic agents, Antihypertensive agents, Lipid-lowering agents;

Model 3^a^: further adjusted for SBP, eGFR, hs-CRP, FBG, LDL-c, Antidiabetic agents, Antihypertensive agents, Lipid-lowering agents, cumCVAI_06 − 10_;

cumCVAI_06 − 10_: cumulative Chinese visceral adiposity index between 2006 and 2010;

cumCVAI_10 − 14_: cumulative Chinese visceral adiposity index between 2010 and 2014

When the combined effect of cumCVAI and early versus late measurement was considered, subjects with cumCVAI ≥ median and a positive slope had the highest CVD risk (HR 1.22, 95% CI 1.03–1.46) when compared with those with a negative slope and cumCVAI < median.

### Hierarchical analyses and sensitivity analyses

Initially, we observed significant interactions between age (≤ 60 years vs. >60 years) and cumCVAI, TWA-cumCVAI, cumulative burden, duration of high CVAI exposure, and early versus late cumCVAI measurement (*P* for interaction < 0.05). The relative risk of CVD was higher for those aged ≤ 60 years. Additionally, even though these associations were not significantly different in the sex and blood pressure attainment status subgroups (*P* for interaction > 0.05), the relative CVD risk might be higher among men and those who did not achieve optimal blood pressure status (Table 4**).**

**Table 4:** Hazard ratios for the outcomes by cumulative CVAI indicators stratified by age, sex, and blood pressure target attainment

	age		sex		blood pressure target attainment	
CumCVAI						
**Index**	**Age < 60**	**Age ≥ 60**	**Male**	**Female**	**No**	**Yes**
**Quartile 1**	Reference	Reference	Reference	Reference	Reference	Reference
**Quartile 2**	1.19(0.92–1.54)	1.17(0.90–1.52)	1.16(0.96–1.41)	1.78(0.96–3.31)	1.16(0.95–1.41)	1.32(0.86–2.04)
**Quartile 3**	1.52(1.16–1.98)	1.16(0.90–1.50)	1.24(1.02–1.50)	1.40(0.74–2.66)	1.23(1.01–1.50)	1.36(0.87–2.12)
**Quartile 4**	1.54(1.20–1.98)	1.14(0.88–1.46)	1.37(1.14–1.65)	1.81(0.95–3.43)	1.30(1.07–1.58)	1.43(0.90–2.27)
***P*** **for interaction**	0.10		0.19		0.50	
**TWA-CVAI**						
**Quartile 1**	Reference	Reference	Reference	Reference	Reference	Reference
**Quartile 2**	1.05(0.81–1.38)	1.06(0.83–1.36)	1.08(0.89–1.30)	1.30(0.71–2.38)	1.06(0.87–1.29)	1.07(0.68–1.67)
**Quartile 3**	1.59(1.22–2.07)	1.07(0.84–1.35)	1.25(1.04–1.51)	1.43(0.79–2.60)	1.23(1.01–1.49)	1.40(0.91–2.16)
**Quartile 4**	1.66(1.29–2.13)	1.10(0.86–1.40)	1.36(1.13–1.63)	1.60(0.87–2.95)	1.30(1.08–1.58)	1.41(0.90–2.23)
***P*** **for interaction**	< 0.01		0.74		0.42	
**Cumulative burden**						
**= 0**	Reference	Reference	Reference	Reference	Reference	Reference
**> 0**	1.57(1.31–1.88)	1.05(0.90–1.23)	1.24(1.09–1.41)	1.35(0.92–1.97)	1.23(1.08–1.40)	1.31(0.96–1.79)
***P*** **for interaction**	< 0.01		0.53		0.22	
**Time exposure duration**						
**0 year**	Reference	Reference	Reference	Reference	Reference	Reference
**2 years**	1.07(0.79–1.45)	1.29(0.97–1.71)	1.24(0.99–1.54)	1.06(0.58–1.94)	1.23(0.98–1.53)	1.26(0.76–2.09)
**4 years**	1.28(0.95–1.74)	1.05(0.78–1.41)	1.18(0.94–1.48)	1.29(0.68–2.41)	1.19(0.95–1.50)	1.27(0.75–2.15)
**6 years**	1.68(1.26–2.25)	1.17(0.88–1.55)	1.43(1.15–1.78)	1.09(0.60–1.98)	1.36(1.09–1.70)	1.66(1.00-2.76)
**Index**	**age**		**sex**		**blood pressure target attainment**	
	**Age < 60**	**Age ≥ 60**	**Male**	**Female**	**No**	**Yes**
**8 years**	1.65(1.17–2.31)	1.18(0.89–1.57)	1.46(1.17–1.82)	1.20(0.68–2.11)	1.37(1.10–1.71)	1.77(1.05–2.99)
**10 years**	1.79(1.33–2.42)	1.25(0.92–1.68)	1.46(1.14–1.86)	1.26(0.72–2.23)	1.39(1.09–1.76)	1.39(0.77–2.53)
***P*** **for interaction**	0.02		0.95		0.59	
**Combination of CumCVAI and CVAI slope**						
**CumCVAI < median Slope < 0**	Reference	Reference	Reference	Reference	Reference	Reference
**CumCVAI < median Slope ≥ 0**	0.97(0.81–1.17)	0.92(0.71–1.18)	1.04(0.86–1.26)	0.70(0.39–1.26)	0.95(0.78–1.16)	1.28(0.81–2.02)
**CumCVAI ≥ median, Slope < 0**	1.17(0.96–1.42)	0.97(0.76–1.25)	1.16(0.94–1.42)	0.73(0.38–1.41)	1.07(0.87–1.32)	1.44(0.85–2.44)
**CumCVAI ≥ median, Slope ≥ 0**	1.23(1.04–1.47)	1.00(0.80–1.26)	1.27(1.05–1.52)	0.84(0.48–1.48)	1.19(1.00-1.44)	1.35(0.85–2.15)
***P*** **for interaction**	0.06		0.63		0.51	

Model 1: adjusted for age and sex;

Model 2: adjusted for age, sex, smoking status, drinking status, education, and physical activity;

Model 3: further adjusted for SBP, eGFR, hs-CRP, FBG, LDL-c, Antidiabetic agents, Antihypertensive agents, Lipid-lowering agents;

The association of cumCVAI with CVD risk was essentially unchanged after performing the Fine–Gray competing risks analysis excluding the subjects who developed CVD with the period of follow-up being less than 1 year (n = 275), the subjects who did not undergo five consecutive examinations from 2006 to 2014 (n = 3,625), and subjects with hepatitis (n = 747) **(**Table 5**).**

**Table 5:** Sensitivity analyses for the association between cumulative CVAI indicators and CVD.

Index	Sensitivity analysis^a^	Sensitivity analysis^b^	Sensitivity analysis^c^	Sensitivity analysis^d^
**CumCVAI**				
**Quartile 1**	Reference	Reference	Reference	Reference
**Quartile 2**	1.21(1.01–1.45)	1.16(0.96–1.41)	1.31(1.06–1.62)	1.19(0.99–1.44)
**Quartile 3**	1.29(1.07–1.54)	1.25(1.03–1.52)	1.41(1.14–1.74)	1.30(1.08–1.57)
**Quartile 4**	1.35(1.13–1.61)	1.33(1.10–1.61)	1.42(1.15–1.74)	1.36(1.14–1.63)
**TWA-CVAI**				
**Quartile 1**	Reference	Reference	Reference	Reference
**Quartile 2**	1.08(0.90–1.30)	1.07(0.88–1.31)	1.09(0.88–1.35)	1.06(0.88–1.28)
**Quartile 3**	1.26(1.06–1.51)	1.27(1.05–1.54)	1.34(1.09–1.65)	1.27(1.06–1.52)
**Quartile 4**	1.35(1.13–1.61)	1.35(1.12–1.62)	1.49(1.22–1.82)	1.35(1.14–1.61)
**Cumulative burden of CVAI**				
**= 0**	Reference	Reference	Reference	Reference
**> 0**	1.24(1.10–1.40)	1.27(1.11–1.44)	1.32(1.15–1.52)	1.26(1.12–1.43)
**Time exposure duration**				
**0 year**	Reference	Reference	Reference	Reference
**2 years**	1.21(0.98–1.48)	1.30(1.04–1.62)	1.28(1.00-1.63)	1.23(1.00-1.52)
**4 years**	1.17(0.95–1.44)	1.13(0.89–1.42)	1.19(0.92–1.53)	1.19(0.96–1.47)
**6 years**	1.38(1.13–1.70)	1.39(1.12–1.73)	1.40(1.10–1.79)	1.39(1.13–1.71)
**8 years**	1.41(1.14–1.73)	1.42(1.14–1.78)	1.46(1.14–1.87)	1.43(1.14–1.79)
**10 years**	1.42(1.14–1.78)	1.45(1.14–1.84)	1.47(1.15–1.87)	1.45(1.18–1.79)
**Index**	**Sensitivity analysis** ^a^	**Sensitivity analysis** ^b^	**Sensitivity analysis** ^c^	**Sensitivity analysis** ^**d**^
**Combination of CumCVAI and CVAI slope**				
**CumCVAI < median, Slope < 0**	Reference	Reference	Reference	Reference
**CumCVAI < median, Slope ≥ 0**	1.01(0.84–1.22)	1.07(0.87–1.30)	1.07(0.87–1.30)	1.02(0.84–1.23)
**CumCVAI ≥ median, Slope < 0**	1.11(0.91–1.35)	1.15(0.93–1.42)	1.15(0.93–1.42)	1.13(0.93–1.38)
**CumCVAI ≥ median, Slope ≥ 0**	1.22(1.03–1.46)	1.28(1.06–1.55)	1.28(1.06–1.55)	1.26(1.06–1.51)

a Sensitivity analysis was performed using competing risk model considering death as a competing risk

b Sensitivity analysis was performed by excluding outcome within the first year of follow-up

c Sensitivity analysis was performed by attending consecutive health-check in 2006–2014 survey

d Sensitivity analysis was performed by excluding participants with history of hepatitis at baseline

Model 1: adjusted for age and sex;

Model 2: adjusted for age, sex, smoking status, drinking status, education, and physical activity;

Model 3: further adjusted for SBP, eGFR, hs-CRP, FBG, LDL-c, Antidiabetic agents, Antihypertensive agents, Lipid-lowering agents;

### Predictive value of CVAI and cumCVAI for new-onset CVD

Changes in the C-index, IDI, and NRI were used to compare the accuracy between CVAI and cumCVAI, which are based on the conventional risk factors for CVD. When adding the cumCVAI to the conventional model, the values of IDI, NRI, and C-index were 0.0005 (0.0001–0.0009; *P* = 0.01), 0.1642 (0.1053–0.2231; *P* < 0.01), and 0.6573 (0.6428–0.671; *P* < 0.01), respectively (Table S2). Compared with the CVAI, the IDI, C-index, and NRI improved after adding cumCVAI to the conventional model.

## Discussion

In this prospective study, a high cumCVAI accelerated the development of adverse CVD-associated events. In particular, early cumulative exposures were substantially higher than those in later years. Additionally, a dependent association between high cumCVAI and CVD risk may exist for age, sex, and optimal blood pressure control. Interestingly, the predictive value of long-term monitoring of dynamic changes in CVAI compared to baseline CVAI is potentially more significant for CVD risk.

Similar to the findings of the current study, which demonstrated that CVAI is closely related to CVD development [14, 28, 29], one previous cross-sectional study regarded CVAI as a predictor of the incidence of CVD [14]. Wang et al. [28] studied a cohort of 9,280 Chinese women with up to 6.59 years of follow-up and found that a high CVD risk was associated with a high baseline CVAI. The current study differs from previous observational studies [30, 31], which involved only a single CVAI measurement in the general population. In this study, an increased risk of CVD was associated with a high cumCVAI among patients with hypertension during the observation window of up to 8 years. Compared with those in quartile 1, the CVD risk was highest in individuals in quartile 4, with an adjusted HR (95% CI) of 1.35 (1.13–1.61). Moreover, with additional adjustment for baseline CVAI, the risk could not attenuate the effect (HR 1.27, 95% CI 1.07–1.50). The long-term cumulative effect appears to be superior to the baseline CVAI regarding the development of new-onset CVD. Thus, automatic extraction of useful information from electronic medical records and the automatically generated indicator, CVAI, which is an important screening indicator, are of great significance and value for patients with hypertension who are at a high risk of developing CVD. Given that electronic medical records allow the rapid integration of data across multiple time points, using these records can provide effective evidence for CVD prevention.

The present study demonstrated that cumCVAI is an independent risk factor for CVD and that the effects on CVD risk at different stages during the observation window are not universally identical. Compared with late measurement (HR 1.24, 95% CI 1.04–1.49), the magnitude of the association appeared more prominent with cumCVAI in earlier life (HR 1.33, 95% CI 1.11–1.59). The results showed lower CVAI values later in the observation period, even when the values were low enough to result in the same accumulation at the same time point. This finding indicates that the lifetime risk cannot be fully reversed with earlier in life. Previous Mendelian randomization studies [32, 33] and the CARDIA cohort study [21] in the US derived a similar conclusion, suggesting that early exposure to elevated LDL-c is associated with significant adverse CVD events later in life. These studies underscore the significance of achieving optimal CVAI early in life.

Notably, given the combined effect of cumCVAI and its time course, the results indicate that the CVD risk increased by 22% in subjects with cumCVAI ≥ median and a positive slope when compared with the reference group. Thus, the risk does not just rely on the magnitude of cumCVAI, but also its time course. Therefore, this study proposes that slope variation and exposure stage can be quantified by time course in the modulation of CVD risk [34]. Future basic experiments, clinical trials, and other related studies on how to quantify the time course will provide potential reference values.

In addition to the above result, the association between high cumCVAI and CVD risk was age-dependent in subjects with hypertension, and was significantly higher in participants aged < 60 years. Two previous studies have reported that even with the same exposure to risk factors, the risk of developing CVD in patients aged < 60 years is higher than in the elderly population, which is in accordance with previous studies reported by Qiao et al. [35] and Cai et al. [30]. Furthermore, this research found a positive association between cumCVAI and CVD risk among men who did not achieve optimal blood pressure control (DBP ≥ 90 mmHg and/or SBP ≥ 140 mmHg), although there was no significant interaction. This finding indicates that achieving early and prolonged optimal blood pressure control among patients with hypertension protects from CVD risk. This intervention provides a strong basis for reducing CVD risk in high-risk individuals with high CVAI values and hypertension, particularly men.

This study demonstrated that fluctuations in the CVAI over time were associated with CVD; however, the exact physiological mechanism explaining the association between CVAI and CVD remains unclear. This research speculates that this phenomenon might be related to the following. First, previous studies have indicated that the CVAI can be used as a simple and reliable surrogate measure of visceral fat in Asian populations [14, 36], which is closely associated with CVD risk. Visceral fat is generally considered “sick fat” and may have a crucial effect on the development of metabolic diseases, such as CVD and insulin resistance [37, 38]. Second, chronic inflammation has been highlighted as a mediator that can accelerate the progression of CVD outcomes associated with visceral fat accumulation. Visceral fat produces various cytokines and proinflammatory hormones, which may lead to a pro-inflammatory response and induce endothelial injury [39, 40]. Similarly, in this study, hs-CRP was higher in the high cumCVAI group than in the low cumCVAI group, and was likely to be associated with a high CVD risk, further verifying the accuracy of the results. Third, in addition to inherited genetic factors, we found that individuals in the high cumCVAI group had high SBP, DBP, FBG, LDL-c, and BMI values, and the majority were alcohol drinkers or smokers, which are primary CVD risk factors. However, biologically meaningful effects might not have been eliminated, even with adjustment for the above confounders. Thus, the risk of CVD was obviously elevated, which could have been due to the synergistic effects of these factors.

The present findings have important value for the prevention and management of CVD among patients with hypertension. Currently, the CVAI is widely used clinically as a surrogate indicator of visceral obesity, further refining the assessment of CVD risk. More attention should be given to the long-term hazards associated with the cumulative exposure and long duration of high CVAI values, rather than focusing on only a single CVAI measurement during routine clinical evaluation. Using data from electronic medical records, CVAI values were automatically generated from traditional indicators, and we utilized repeated measurement data at different time points to capture the dynamic cumulative changes. Thus, measures for implementing electronic medical record information management and popularizing personal dynamic monitoring devices will provide future directions for the primary prevention of CVD. Importantly, considering that the lifetime CVD risk depends on early cumulative exposure to risk factors, identification of high-risk individuals and timely intervention to reduce cardiovascular symptoms and events has relevant practical implications.

This study has some notable strengths. First, the data used in this research were obtained from a large, longitudinal, and well-designed cohort to evaluate the association between cumulative CVAI exposure and its time course on the risk of developing adverse outcomes among patients with hypertension. Moreover, the Kailuan Study adopted a standardized protocol for multiple potential confounders, including anthropometric measurements, lifestyle behaviors, and laboratory indicators, to ensure the quality of data collection.

Despite these strengths, there are still limitations in this research. First, although as much data as possible were collected for potential confounders, other relevant factors, such as environmental changes, were not considered owing to the limitations of the study design. Second, this study used an urban agglomeration from northern China as the research cohort; therefore, the results may not be completely generalizable to other populations. However, the homogeneity of the population in this study lends reliability to the results. Third, CVAI values may be subject to measurement bias. Additionally, MRI and CT, the gold-standard techniques for assessing visceral fat, were not used to verify coherence with the actual amount of visceral fat and the CVAI values owing to the large sample size and limited research funds.

## Conclusions

In summary, this study implies that incident CVD risk relies on both high cumCVAI exposure, and even more so on the long-term duration of high CVAI exposure among patients with hypertension. Furthermore, this risk is considered to be regulated by the time course of CVAI accumulation (i.e., early exposure). Thus, close long-term monitoring of dynamic CVAI changes and early control of lipid metabolism could provide a theoretical basis for CVD event prevention among patients with hypertension. The current study suggests that the CVAI should be tightly controlled to achieve comprehensive cardiovascular benefit.

## Electronic supplementary material

Below is the link to the electronic supplementary material.

Supplementary Material 1

Supplementary Material 2

## Data Availability

The datasets supporting the conclusions of this article are available in the [repository name] repository, [unique persistent identifier and hyperlink to dataset(s) in http:// format].

## Abbreviations

CVAI: Chinese visceral adiposity index
CVD: Cardiovascular disease
TG: Triglyceride
FBG: Fasting blood glucose
MI: Myocardial infarction
HDL-c: High-density lipoprotein cholesterol
LDL-c: Low-density lipoprotein cholesterol
hs-CRP: High-sensitivity C-reactive protein
TC: Total cholesterol
SBP: Systolic blood pressure
DBP: Diastolic blood pressure
eGFR: Estimated glomerular filtration rate

## References

[1] ROTH G A, MENSAH G A, JOHNSON C O (2020). Global Burden of Cardiovascular Diseases and Risk factors, 1990–2019: Update from the GBD 2019 study [J]. J Am Coll Cardiol.

[2] JOSEPH P, LEONG D (2017). Reducing the Global Burden of Cardiovascular Disease, Part 1: the epidemiology and risk factors [J]. Circ Res.

[3] KRIST A H, DAVIDSON K W, MANGIONE C M (2020). Behavioral counseling interventions to promote a healthy Diet and Physical Activity for Cardiovascular Disease Prevention in adults with Cardiovascular Risk factors: US Preventive Services Task Force Recommendation Statement [J]. JAMA.

[4] KIM MS, KIM W J, KHERA A V (2021). Association between adiposity and cardiovascular outcomes: an umbrella review and meta-analysis of observational and mendelian randomization studies [J]. Eur Heart J.

[5] FUCHS F D, WHELTON PK (2020). High blood pressure and Cardiovascular Disease [J]. Hypertension.

[6] CHEN MQ, SHI W R, WANG H Y (2021). Sex differences of combined Effects between hypertension and general or central obesity on ischemic stroke in a middle-aged and Elderly Population [J]. Clin Epidemiol.

[7] HUANG Y, LIU Y, MA Y (2022). Associations of visceral adipose tissue, circulating protein biomarkers, and risk of Cardiovascular Diseases: a mendelian randomization analysis [J]. Front Cell Dev Biol.

[8] WEI J, LIU X. XUE H, Comparisons of visceral Adiposity Index, body shape index, body Mass Index and Waist circumference and their Associations with Diabetes Mellitus in adults [J]. Nutrients, 2019, 11(7). 10.3390/nu11071580 PMC6683101 31336951

[9] HAN M, QIE R, LI Q (2021). Chinese visceral adiposity index, a novel indicator of visceral obesity for assessing the risk of incident hypertension in a prospective cohort study [J]. Br J Nutr.

[10] BRAHIMAJ A, RIVADENEIRA F, MUKA T (2019). Novel metabolic indices and incident type 2 diabetes among women and men: the Rotterdam study [J]. Diabetologia.

[11] HAN M, QIN P, LI Q (2021). Chinese visceral adiposity index: a reliable indicator of visceral fat function associated with risk of type 2 diabetes [J]. Diabetes Metab Res Rev.

[12] YU J, YI Q, CHEN G (2022). The visceral adiposity index and risk of type 2 diabetes mellitus in China: a national cohort analysis [J]. Diabetes Metab Res Rev.

[13] WU J, GONG L, LI Q (2017). A Novel Visceral Adiposity Index for Prediction of type 2 diabetes and pre-diabetes in chinese adults: a 5-year prospective study [J]. Sci Rep.

[14] WAN H, WANG Y, XIANG Q (2020). Associations between abdominal obesity indices and diabetic complications: chinese visceral adiposity index and neck circumference [J]. Cardiovasc Diabetol.

[15] ZHAO M, SONG L, SUN L (2021). Associations of type 2 diabetes Onset Age with Cardiovascular Disease and Mortality: the Kailuan study [J]. Diabetes Care.

[16] WANG X, FENG B, HUANG Z (2022). Relationship of cumulative exposure to the triglyceride-glucose index with ischemic stroke: a 9-year prospective study in the Kailuan cohort [J]. Cardiovasc Diabetol.

[17] ZHANG Y, PLETCHER M J VITTINGHOFFE (2021). Association between cumulative low-density lipoprotein cholesterol exposure during Young Adulthood and Middle Age and Risk of Cardiovascular events [J]. JAMA Cardiol.

[18] TIAN X, WANG A, ZUO Y (2022). Time course of serum uric acid accumulation and the risk of diabetes mellitus [J]. Nutr Diabetes.

[19] KANG K, WANG Y, WU J (2020). Association between cumulative exposure to increased low-density lipoprotein cholesterol and the prevalence of asymptomatic intracranial atherosclerotic stenosis [J]. Front Neurol.

[20] CUI H, LIU Q, WU Y (2022). Cumulative triglyceride-glucose index is a risk for CVD: a prospective cohort study [J]. Cardiovasc Diabetol.

[21] DOMANSKI MJ, TIAN X, WU C O (2020). Time course of LDL cholesterol exposure and Cardiovascular Disease Event risk [J]. J Am Coll Cardiol.

[22] ZHAO M, DU W, ZHAO Q (2022). Transition of metabolic phenotypes and risk of Atrial Fibrillation according to BMI: kailuan study [J]. Front Cardiovasc Med.

[23] WANG L, LEE Y, WU Y (2021). A prospective study of waist circumference trajectories and incident cardiovascular disease in China: the Kailuan Cohort Study [J]. Am J Clin Nutr.

[24] CHOBANIAN A V, BAKRIS G L, BLACK H R (2003). Seventh report of the Joint National Committee on Prevention, detection, evaluation, and treatment of high blood pressure [J]. Hypertension.

[25] The Prevention of Diabetes Mellitus [J] (2021). JAMA.

[26] LI W, JIN C, VAIDYA A (2017). Blood pressure trajectories and the risk of Intracerebral Hemorrhage and cerebral infarction: a prospective study [J]. Hypertension.

[27] JIN C, CHEN S, VAIDYA A (2017). Longitudinal change in fasting blood glucose and myocardial infarction risk in a Population without diabetes [J]. Diabetes Care.

[28] WANG Y, ZHAO X, CHEN Y (2022). Visceral adiposity measures are strongly associated with cardiovascular disease among female participants in Southwest China: a population-based prospective study [J]. Front Endocrinol (Lausanne).

[29] XIE X, LI Q, ZHANG L, Lipid accumulation product, visceral adiposity index, and chinese visceral adiposity index as markers of cardiometabolic risk in adult growth hormone deficiency patients. : a cross-sectional study [J]. Endocr Pract, 2018, 24(1): 33 – 9. 10.4158/EP-2017-0007 29144802

[30] CAI X, LI N (2022). Nonlinear relationship between chinese visceral Adiposity Index and New-Onset myocardial infarction in patients with hypertension and obstructive sleep apnoea: insights from a Cohort study [J]. J Inflamm Res.

[31] LI B, LAI X (2020). The associations between neutrophil-to-lymphocyte ratio and the chinese visceral Adiposity Index, and carotid atherosclerosis and atherosclerotic cardiovascular disease risk [J]. Exp Gerontol.

[32] FERENCE B A, KASTELEIN J J P, GINSBERG H N (2017). Association of genetic variants related to CETP inhibitors and statins with lipoprotein levels and Cardiovascular risk [J]. JAMA.

[33] FERENCE B A, MAJEED F, PENUMETCHA R (2015). Effect of naturally random allocation to lower low-density lipoprotein cholesterol on the risk of coronary heart disease mediated by polymorphisms in NPC1L1, HMGCR, or both: a 2 × 2 factorial mendelian randomization study [J]. J Am Coll Cardiol.

[34] VANDERWEELE T J, JACKSON J W LIS (2016). Causal inference and longitudinal data: a case study of religion and mental health [J]. Soc Psychiatry Psychiatr Epidemiol.

[35] QIAO T, LUO T, PEI H (2022). Association between abdominal obesity indices and risk of cardiovascular events in chinese populations with type 2 diabetes: a prospective cohort study [J]. Cardiovasc Diabetol.

[36] XIA M F, CHEN Y, LIN HD (2016). A indicator of visceral adipose dysfunction to evaluate metabolic health in adult chinese [J]. Sci Rep.

[37] BARCHETTA I, CIMINI F A, CICCARELLI G (2019). Sick fat: the good and the bad of old and new circulating markers of adipose tissue inflammation [J]. J Endocrinol Invest.

[38] BASTIEN M, POIRIER P, LEMIEUX I (2014). Overview of epidemiology and contribution of obesity to cardiovascular disease [J]. Prog Cardiovasc Dis.

[39] BASTARD JP, MAACHI M, LAGATHU C (2006). Recent advances in the relationship between obesity, inflammation, and insulin resistance [J]. Eur Cytokine Netw.

[40] DU T, YUAN G, ZHANG M (2014). Clinical usefulness of lipid ratios, visceral adiposity indicators, and the triglycerides and glucose index as risk markers of insulin resistance [J]. Cardiovasc Diabetol.

