# Supplementary material for: Association of the time course of Chinese visceral adiposity index accumulation with cardiovascular events in patients with hypertension
Source: Lipids Health Dis. 2023 Jul 1;22:90. doi: 10.1186/s12944-023-01852-w (PMC10314383; doi:10.1186/s12944-023-01852-w)
Supplement: Supplementary file 1 — Supplementary Material 1 [file 12944_2023_1852_MOESM1_ESM.docx]

| **A.** | **B.** | | **C.** |
| --- | --- | --- | --- |
| **D.**  **log-rank p<0.01** | | **E.**  **log-rank p<0.01** | |

**Supplementary Fig. 1** Kaplan–Meier curves of incidence of outcomes according to the different categories of CVAI. **A** CumCVAI; **B** TWA-CVAI; **C** Cumulative burden ;D Time exposure duration;

E Combination of CumCVAI and CVAI slope

Combination of CumCVAI and CVAI slope
