# Supplementary material for: Association of the time course of Chinese visceral adiposity index accumulation with cardiovascular events in patients with hypertension
Source: Lipids Health Dis. 2023 Jul 1;22:90. doi: 10.1186/s12944-023-01852-w (PMC10314383; doi:10.1186/s12944-023-01852-w)
Supplement: Supplementary file 2 — Supplementary Material 2 [file 12944_2023_1852_MOESM2_ESM.docx]

| **Supplementary Table 1** Hazards ratios and 95% confidence interval for the risk of CVD stratified by cumulative CVAI indicators | | | | | |
| --- | --- | --- | --- | --- | --- |
| **Index** | **Quartile 1** | **Quartile 2** | **Quartile 3** | **Quartile 4** | ***P* for trend** |
| **CumCVAI** |  | | | |  |
| **Case, n (%)** | 204（5.32） | 278（7.24） | 337（8.78） | 365（9.51） |  |
| **Incidence rate^a^** | 8.23（7.17-9.44） | 11.55（10.27-12.99） | 14.40（12.94-16.03） | 16.03（14.46-17.76） |  |
| **Model 3** | Reference | 1.21（1.01-1.45） | 1.29（1.07-1.54） | 1.35（1.13-1.61） | <0.01 |
| **Model 3^b^** | Reference | 1.17（0.97-1.41） | 1.18（0.94-1.47） | 1.28（1.06-1.56） | <0.01 |
| **Model 3^c^** | Reference | 1.18（0.98-1.42） | 1.21（0.98-1.49） | 1.30（1.08-1.57） | <0.01 |
| **TWA-CVAI** | | | | | |
| **Case, n (%)** | 213（5.55） | 259（6.75） | 346（9.02） | 366（9.54） |  |
| **Incidence rate^a^** | 8.73（7.63-9.98） | 10.82（9.58-12.22） | 14.73（13.25-16.36） | 15.79（14.25-17.49） |  |
| **Model 3** | Reference | 1.09（0.91-1.31） | 1.28（107-1.52） | 1.35（1.14-1.61） | <0.01 |
| **Model 3^b^** | Reference | 1.07（0.88-1.29） | 1.20（0.96-1.49） | 1.30（1.08-1.58） | <0.01 |
| **Model 3^c^** | Reference | 1.07（0.89-1.29） | 1.22（0.99-1.50） | 1.32（1.10-1.59） | <0.01 |

Model 3：further adjusted for SBP, eGFR, hs-CRP, FBG, LDL-c, Antidiabetic agents, Antihypertensive agents, Lipid-lowering agents;

Model 3^b^：further adjusted for SBP, eGFR, hs-CRP, FBG, LDL-c, Antidiabetic agents, Antihypertensive agents, Lipid-lowering agents, CVAI_06_;

Model 3^c^：further adjusted for SBP, eGFR, hs-CRP, FBG, LDL-c, Antidiabetic agents, Antihypertensive agents, Lipid-lowering agents, CVAI_14_;

| **Supplementary Table 2** Incremental ability of the CVAI and cumulative CVAI (cumCVAI) to predict CVDs | | | | | | |
| --- | --- | --- | --- | --- | --- | --- |
| **Index** | **C-index** | | **IDI** | | **Category-free NRI** | |
|  | **Est.(95%CI)** | ***P*** | **Est.(95%CI)** | ***P*** | **Est.(95%CI)** | ***P*** |
| Clinical risk factors | 0.6549(0.6404-0.6694) | - | - | - | - | - |
| Clinical risk factors+CVAI | 0.6571(0.6426-0.6716) | <0.01 | 0.0003(0.0001-0.0006) | 0.05 | 0.1315(0.0725-0.1904) | <0.01 |
| Clinical risk factors+cumCVAI | 0.6573(0.6428-0.6719) | <0.01 | 0.0005(0.0001-0.0009) | 0.01 | 0.1642(0.1053-0.2231) | <0.01 |

Clinical risk factors: age, sex, smoking, drinking, education level, salt status, physical activity, SBP, eGFR, hs-CRP, FBG, LDL-c, antidiabetic drugs, antihypertensive drugs and lipidlowering drugs
